# Plyometric Training Practices of Brazilian Olympic Sprint and Jump Coaches: Toward a Deeper Understanding of Their Choices and Insights

**DOI:** 10.5114/jhk/169167

**Published:** 2023-07-15

**Authors:** Irineu Loturco, Lucas A. Pereira, Tomás T. Freitas, Túlio B. M. A. Moura, Valter P. Mercer, Victor Fernandes, Neilton S. A. Moura, Nélio A. Moura, Adam Zając, Chris Bishop

**Affiliations:** 1 NAR—Nucleus of High Performance in Sport, São Paulo, Brazil.; 2 Department of Human Movement Sciences, Federal University of São Paulo, São Paulo, Brazil.; 3 University of South Wales, Pontypridd, Wales, United Kingdom.; 4 UCAM Research Center for High Performance Sport, UCAM Universidad Católica de Murcia, Murcia, Spain.; 5 Facultad de Deporte, UCAM Universidad Católica de Murcia, Murcia, Spain.; 6 UNG—University of Guarulhos, São Paulo, Brazil.; 7 Pinheiros Sport Club, São Paulo, Brazil.; 8 Chinese Athletics Association, Beijing, China.; 9 Institute of Sport Sciences, The Jerzy Kukuczka Academy of Physical Education in Katowice, Katowice, Poland.; 10 London Sport Institute, Middlesex University, London, United Kingdom.

**Keywords:** athletic performance, muscle power, speed, track and field, jumping

## Abstract

Plyometric training is extensively used by coaches to enhance neuromuscular performance in a wide variety of sports. Due to the high demands of sprint speed and power output in elite sprinters and jumpers, sprint and jump coaches are likely to have great knowledge on this topic. Undoubtedly, this expertise is even more pronounced for Olympic coaches, who work with some of the fastest and most powerful athletes in the world, and who are required to continually maintain these athletes at optimal performance levels. Describing and discussing the practices commonly adopted by these coaches in detail and extrapolating this experience to other sport coaching contexts and disciplines may be extremely relevant. The current article presents, explores, and illustrates the plyometric training practices of Brazilian Olympic sprint and jump coaches, with a special focus on training programming and exercise selection.

## Introduction

Plyometric exercises are widely used by coaches from numerous sports, during different phases of the annual training season (Bolger et al., 2016; Loturco et al., 2022; Weldon et al., 2022a, 2022b). In more general terms, plyometrics (or stretch-shortening cycle [SSC] exercises) can be characterized by a rapid transition from the deceleration (e.g., drop landing) to the acceleration phase (e.g., a vertical jump) or by the combination of eccentric and concentric contractions (Fatouros et al., 2000; Hermassi et al., 2010). The efficiency of the SSC to improve the subsequent motor-task (e.g., a maximal vertical or horizontal jump) is well established in the literature and relies on the mechanical properties of the muscle-tendon complex (Flanagan and Comyns, 2008; McMahon, 2018). During plyometric activities, the muscle-tendon complex stores potential elastic energy in the eccentric phase, restoring part of this energy during the concentric portion of the movement (Fouré et al., 2012; Fukashiro et al., 2006; Li et al., 2021; McGuigan et al., 2006; McMahon, 2018). This, in turn, leads to increased force and power production, as a result of the SSC potentiation (Fukashiro et al., 2006; McMahon, 2018; Sole, 2018). Several studies have examined the influence of this enhanced effect on physical performance of athletes from different sports, of different ages and competitive levels, confirming that plyometric training is an efficient and safe strategy to improve speed- and power-related capabilities (Markovic, 2007; Saez de Villarreal et al., 2012).

Specifically in track and field, the sport where we can find the fastest athletes on earth, plyometric exercises are used with the objective of enhancing sprint ability (Whelan et al., 2016). Indeed, two recent surveys with sprint and jump coaches revealed that plyometrics are the most frequently prescribed exercises in their training programs, being considered as one of the most important drills for improving sprinting skills by the vast majority of these coaches (Bolger et al., 2016; Healy et al., 2021). The rationale behind these choices and preferences is based on a number of factors that range from “similarity to sprinting” and “potential performance adaptations” to “specific muscle recruitment” and “practicality” (i.e., minimal equipment needed, low-cost, ease of implementation, time-efficiency, and low risk of injury) (Bolger et al., 2016; Healy et al., 2021; Husbands, 2013; Whelan et al., 2016). For Brazilian Olympic sprint and jump coaches, the main reason for prescribing plyometric training programs for elite sprinters and jumpers is “speed development”, with “improvements in jumping ability” and “lower-body power” being, respectively, the second and third most cited reasons for systematically utilizing plyometric exercises (which is largely expected due to the inherent characteristics of jump-based activities) (Loturco et al., 2023b; Ramirez-Campillo et al., 2020).

The beliefs and perceptions about the importance, efficacy, and applicability of plyometrics are not exclusive to track and field coaches. As previously mentioned, this practical and efficient training strategy is widely used by practitioners from a range of sports (e.g., soccer, rugby, volleyball, and cricket), in both the preparatory and competitive periods (Loturco et al., 2022; Weldon et al., 2021, 2022a; Zabaloy et al., 2022). It is worth noting, however, that despite the evident and marked differences between these sports (i.e., sprint and jump events compared to team-sport disciplines), the primary goal of strength and conditioning coaches when incorporating plyometric drills in their training routines is the same: to optimize the speed and power performance of their athletes (Loturco et al., 2022; Weldon et al., 2022b). In this regard, identifying plyometric training practices adopted by coaches who work with sprinters and jumpers, competitors usually distinguished by their exceptional performance in explosive events (Canata and Casale, 2022; Loturco et al., 2021, 2023a), may be of high practical relevance for elite athletes representing different sport disciplines. In this article, we describe and discuss in detail plyometric training strategies commonly used by Brazilian Olympic sprint and jump coaches, with a special emphasis on how these exercises are programmed, exercise selection, and potential training effects.

### Plyometric Training Programming

In general, plyometric training is used over the entire training season, with different configurations between preparatory and competitive periods (Tables 1, 2, and 3). Most sprint and jump coaches prescribe plyometrics either on alternate days (relative to resistance training), after resistance training sessions or within complex training session designs. It is important to emphasize that plyometric training may be more efficient when applied under well-rested conditions (Chu, 1986; Comfort and Matthews, 2010); thus, coaches from other sports (e.g., team-sport disciplines) should take this information into account when designing plyometric training programs. Sprinters and jumpers usually perform at a superior level in strength-power training sessions and, therefore, track and field coaches may use this sequential arrangement (i.e., resistance training + plyometrics) to maximize training responses and induce greater adaptations via post-activation performance enhancement (Boullosa, 2021; Harrison et al., 2019). For the above-mentioned reasons, athletes from other sports might be more benefited by performing plyometric exercises in isolated training sessions or as part of complex training methods (Cormier et al., 2022; Slimani et al., 2016).

**Table 1:** Typical plyometric training program of an Olympic sprinter during preparatory and competitive periods.

	Preparatory period	Competitive period
Weekly frequency	x 3	x 2
**Exercises** **(Sets x reps)**	Standing jumps (3 x 4) Bounding (4 x 8–10) Box jumps (5 x 5) Drop jumps (3 x 4) Multiple hops (4 x 6) Assisted jumps (6 x 6–8)	Hurdle jumps (3 x 4) Bounding (4 x 8–10) Box jumps (5 x 5) Drop jumps (3 x 4) Multiple hops (4 x 6) Assisted jumps (6 x 6–8)
**Interval between sets/exercises**	2–3 min	3–5 min

**Table 2:** Typical plyometric training program of an Olympic long jumper during preparatory and competitive periods.

	Preparatory period	Competitive period
Weekly frequency	x 3	x 2
**Exercises** **(Sets x reps)**	Bounding (6–8 x 12–20) Assisted jumps (2–6 x 4–6) Box drills (5–10 x 6) Hurdle jumps (5–6 x 6) Multiple hops (4–10 x 3–5) Drop jumps (4–6 x 4–6)	Bounding (4–6 x 8–15) Assisted jumps (2–4 x 4–6) Hurdle jumps (4 x 6) Multiple hops (3–6 x 3–5) Drop jumps (3 x 4)
**Interval between sets/exercises**	2–3 min	3–5 min

**Table 3:** Typical plyometric training program of an Olympic high jumper during preparatory and competitive periods.

	Preparatory period	Competitive period
Weekly frequency	x 2	x 1–2
**Exercises** **(Sets x reps)**	Bounding (1 x 12) Hurdle jumps (6 x 4–5) Multiple hops (3–6 x 4–10) Standing jumps (6 x 3) Drop jumps (2 x 6–8)	Bounding (1 x 10) Assisted jumps (3 x 8) Hurdle jumps (4 x 3) Multiple hops (3 x 3) Standing jumps (3–4 x 2–3)
**Interval between sets/exercises**	2–3 min	3–5 min

Overall, plyometric exercises are divided into different categories, according to the expected (or intended) adaptations. Whereas horizontally-directed jumps (e.g., broad jumps, leg bounding, and standing long jumps) are typically utilized to increase sprint acceleration and horizontal jump distance, vertically-directed jumps with relatively shorter ground contact times (CTs) or with “negative loads” (e.g., in-place pogo jumps, and assisted jumps) may be more recommended to enhance top-speed qualities (Clark, 2018; Loturco et al., 2015b, 2015c; Makaruk et al., 2020). To improve lower-body power performance, SSC function, and leg stiffness, exercises such as drop jumps and hurdle jumps are usually prescribed with different instructions (i.e., jumping for maximum height or minimum CT) and intensities (i.e., varying dropping or hurdle heights), which will directly impact their technical execution and expected training outcomes (Khuu et al., 2015; Oliver et al., 2021; Struzik et al., 2016). These applied concepts, as well as those related to the development of speed and power qualities, may be used by practitioners from different sports, who can select and adjust plyometric training content according to the specific needs and demands of athletes and sport disciplines. The high levels of speed and power performance regularly exhibited by Olympic sprinters and jumpers justify and endorse the use of some of their regular training practices within other sport environments. In the following topics, we will describe and discuss the potential applications, efficacy, and main characteristics of plyometric exercises most commonly prescribed by Brazilian Olympic sprint and jump coaches.

### Plyometric Exercises

The types of plyometric exercises most commonly used by Brazilian Olympic sprint and jump coaches as well as their frequency of utilization are described in Table 4.

**Table 4:** Preference list of plyometric exercises for Brazilian Olympic sprint and jump coaches (ordered by relative frequency of utilization).

Exercise	% Of coaches who use the respective exercise	Weekly frequency of use
Hurdle jumps	89%	1–3 x
Bounding	79%	1–3 x
* Box jumps	74%	2–3 x
Drop jumps	74%	2–3 x
Multiple hops	68%	1–3 x
* Assisted jumps	42%	1–3 x
* Standing jumps	21%	1–2 x

Note:* Box drills, assisted jumps, and standing jumps are not necessarily plyometric activities, especially when executed in a non-sequential (“non-continuous”) manner. However, these exercises are frequently mentioned as types of “plyometric exercises” in a series of surveys conducted with coaches from different sports (Healy et al., 2021; Loturco et al., 2022, 2023; Weldon et al., 2021, 2022).

#### Hurdle Jumps

**Figure 1:**
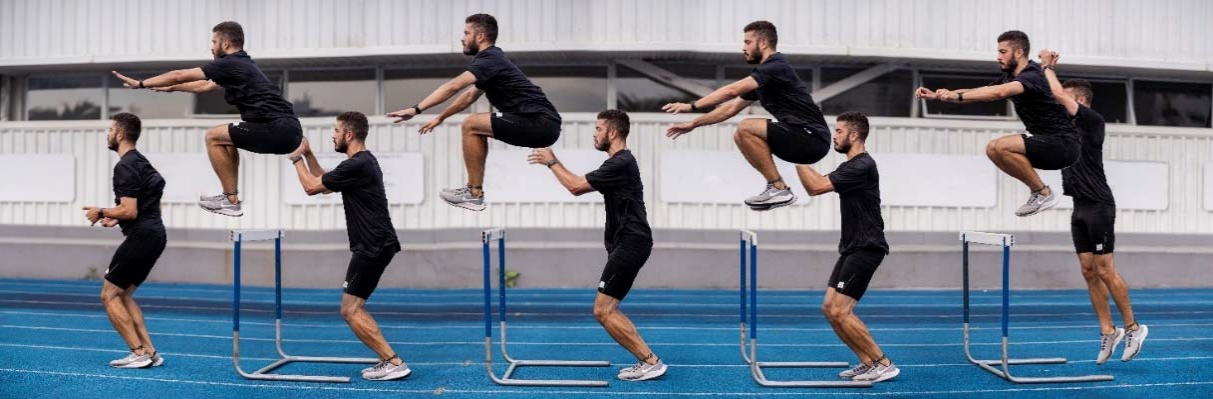
Hurdle jumps sequencing.

The hurdle jump (Figure 1) is unquestionably one of the most popular exercises within track and field environments (Healy et al., 2021; Loturco et al., 2023b). A survey on resistance training practices of sprint coaches revealed that the hurdle jump is not only the most frequently prescribed plyometric drill, but rather, the most commonly used exercise among a variety of traditional, ballistic, and plyometric exercises (Healy et al., 2021). Besides its practicality, efficacy, and cost-effectiveness (which are common characteristics of unloaded jumps), the extensive utilization of hurdle jumps may be favored by the massive presence of hurdles in sports training centers and by the possibility of adjusting hurdle height (and thus, training intensity) from session to session, according to training objectives, and fatigue status (i.e., fatigued or non-fatigued state). Previous studies have shown that jumping forward and sequentially over hurdles may be an optimal strategy for developing speed and power capacities in athletes from different sports (Cappa and Behm, 2011, 2013; Chelly et al., 2010, 2015). A 10-week intervention with young male track athletes revealed that the inclusion of hurdle and depth jumps into their regular training program substantially improved jumping performance and sprint speed over both acceleration (5-m) and top-speed (35–40-m) phases (~10% vs. 5%, 10% vs. 4%, and 4% vs. 2%, for jump, acceleration, and top-speed performance, respectively, relative to a traditional in-season training regimen) (Chelly et al., 2015). Similar results (i.e., superior increases in speed- and power-related capabilities) were observed for soccer players in an 8-week study comparing the effects of a combined training scheme (i.e., biweekly plyometric sessions comprising hurdle and depth jumps added to their usual routine) with customary in-season soccer training (Chelly et al., 2010). The efficacy of hurdle jumps may be related to their mechanical aspects and training specificity (Cappa and Behm, 2011). For example, when compared to the countermovement jump (CMJ), hurdle jumps executed at ~100–140% of the CMJ height exhibit considerably lower CTs and greater ground reaction forces (GRF) (~810 vs. 180 ms and 2150 vs. 4200 N, for CT and GRF of CMJs and hurdle jumps, respectively) (Cappa and Behm, 2011; Dintiman and Ward, 1988). As a matter of comparison, the average CT reported for maximal sprints, bounding, high jumping, long jumping, and directional changes is, respectively, 90, 175, 130, 110, and 250 ms, whereas GRFs described for these activities were > 3100 N (up to ~6100 N) (Cappa and Behm, 2011; Dintiman and Ward, 1988). However, it is important to emphasize that, for this type of the jump, CT increases with increasing hurdle height, which might compromise the effective use of the SSC (Healy et al., 2020). Therefore, practitioners should carefully evaluate athletes’ CT during the execution of hurdle jumps of varying heights, in order to select adequate plyometric training intensities for their athletes (Healy et al., 2020). Providing these recommendations are followed, hurdle jumps can better and more closely reflect the biomechanical characteristics of numerous track and field events and sport-specific activities, which potentially provides a more specific and effective transfer to performance (Cappa and Behm, 2011, 2013; Chelly et al., 2015). Coaches from different sports may consider the inclusion of these practical drills into their regular training routines, bearing in mind that: 1) when CTs are excessively long (i.e., > 250 ms), lower hurdles may be necessary to reduce landing forces and, hence, preserve the efficiency of the fast SSC (i.e., reutilization of elastic energy) (Flanagan and Comyns, 2008; Healy et al., 2020; Jarvis et al., 2022); and 2) a few sets completed twice per week can significantly improve a wide range of sports skills (especially when these exercises are combined with other types of plyometric exercises, e.g., depth jumps).

#### Drop Jumps

**Figure 2:**
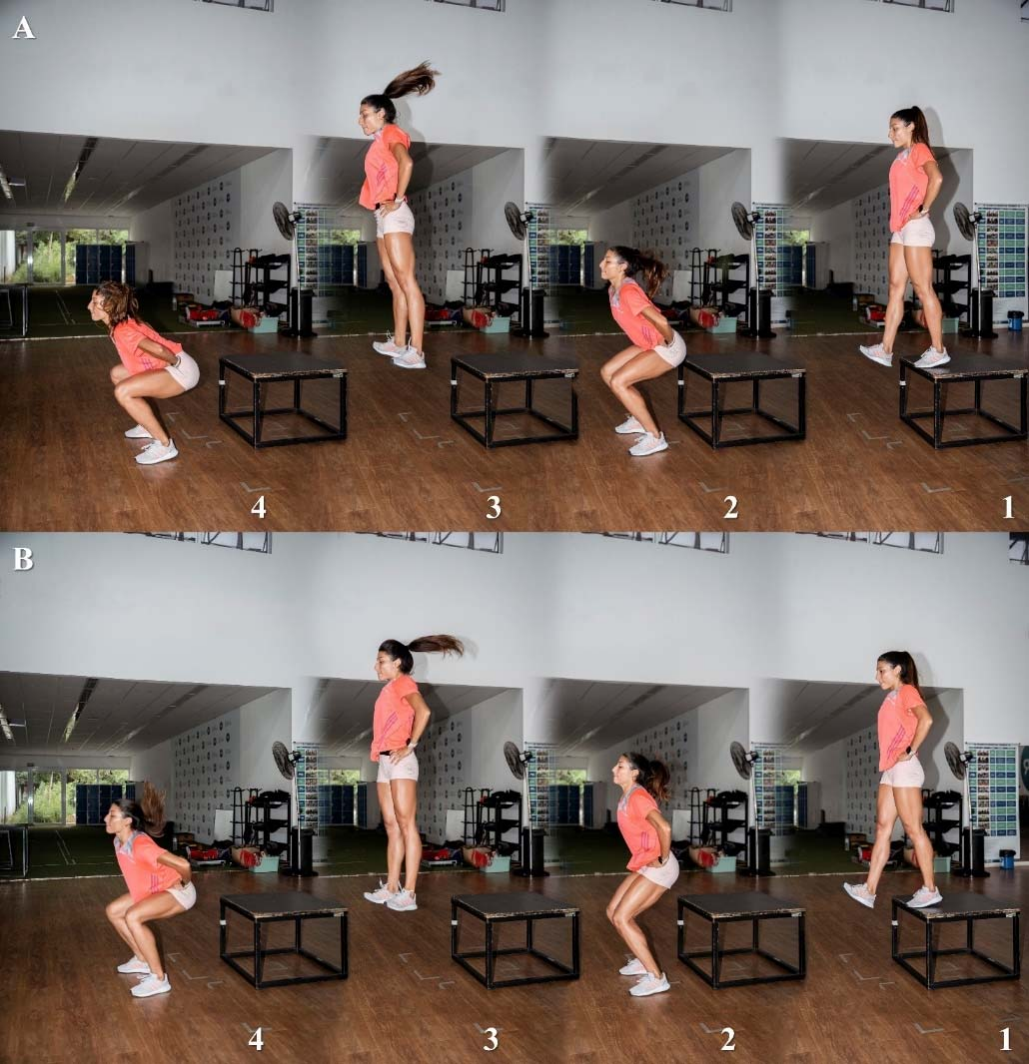
Drop jumps execution, under two distinct verbal instructions: “jumping for maximum height” (Panel A) and “jumping for maximum height with minimum contact time” (Panel B), at the initial (1), contact (2), flight (3), and landing (4) phases.

Drop (or depth) jumps are another example of a practical and efficient plyometric exercise, which also allows the control and adjustment of training intensity by just changing the drop height (Marshall and Moran, 2013; Pedley et al., 2017; Peng et al., 2017). For this reason, practitioners from different sports regularly prescribe drop jumps of various intensities (i.e., different dropping heights) during distinct phases of the season, with different purposes and objectives (e.g., developing sprint speed, jumping ability, running economy) (Healy et al., 2021; Pedley et al., 2017; Weldon et al., 2022b). In track and field practices, the drop jump emerges as one of the most popular training drills, being, for example, the third most frequent type of the jump included in the preference list of Brazilian Olympic sprint and jump coaches (Bolger et al., 2016; Healy et al., 2021; Loturco et al., 2023b; Whelan et al., 2016). Drop jumps are usually performed from plyometric boxes (Figure 2), in an intermittent manner, and with two different verbal instructions: 1) jumping for maximum height, and 2) jumping for maximum height with minimum CT (Khuu et al., 2015; Oliver et al., 2021; Pedley et al., 2017; Struzik et al., 2016). Previous research has consistently shown that these distinct verbal cues can greatly affect the biomechanics and technique of drop jumps, as well as potential training effects (Bobbert et al., 1987; Khuu et al., 2015; Oliver et al., 2021; Pedley et al., 2017; Struzik et al., 2016). Overall, when instructed to minimize CT, athletes typically land stiffer, achieving lower jump heights than, for example, when instructed to jump as high as possible (Khuu et al., 2015). Under this drop jump condition, athletes adopt a more vertically-oriented trunk position, exhibiting lower degrees of hip, knee, and ankle flexion during landing. These biomechanical alterations are also associated with higher levels of ground reaction forces, power production, the rate of force development, and the reactive strength index (Barker et al., 2021; Bobbert et al., 1987; Khuu et al., 2015; Newton et al., 2001; Vanrenterghem et al., 2008; Young et al., 1999), which, from an applied perspective, can lead to conflicting and divergent interpretations. Whereas this increased mechanical efficiency seems to be more closely related to certain sport-specific activities (i.e., linear sprints and rapid jumps) (Douglas et al., 2018; Flanagan and Comyns, 2008; Pedley et al., 2017; Young et al., 1999), high loading magnitudes during the execution of drop jumps may also increase the potential risk of injury, especially when these exercises are performed frequently (Flanagan and Comyns, 2008; Newton et al., 2001; Peng et al., 2011; Sotiropoulos et al., 2023; Young et al., 1999). On the other hand, when jumping for maximum height, athletes display greater trunk, hip, knee, and ankle flexion, a movement pattern that is typically associated with longer ground CTs (i.e., landing + push-off phases) and greater jump heights (an average increase of ~10–20%, compared to “upright jumping”) (Khuu et al., 2015; Oliver et al., 2021; Pedley et al., 2017; Vanrenterghem et al., 2008). In fact, the restriction of the trunk inclination that naturally occurs when athletes are instructed to minimize ground CT will lead to increased torque and power output in the knee joint, with a simultaneous reduction in hip joint torque and power (Vanrenterghem et al., 2008). Since hip jump power is positively correlated with jump performance, a concomitant reduction in vertical jump height is also expected (Áragón-Vargas and Gross, 1997; Lees et al., 2004; Vanrenterghem et al., 2008). These changes in drop jump technique as a result of different verbal cues will certainly impact training-induced adaptations, as highlighted and stated by different authors (Bobbert, 1990; Flanagan and Comyns, 2008; Khuu et al., 2015; Marshall and Moran, 2013; Pedley et al., 2017). For Bobbert (1990), technique is, indeed, the most important variable to be controlled during drop jump training programs. Marshall and Moran (2013) confirmed these assumptions showing that a “countermovement style drop jump program” (i.e., subjects instructed to make a larger countermovement before jumping for maximum height) is more effective than a “bounce style drop jump program” at increasing jump heights (i.e., 6% vs. −0.4%, respectively, for variations in countermovement jump height, after an 8-week training intervention). Young et al. (1999) also found similar results when comparing the effects of two drop jump training methods: 1) “drop jump for maximum rebound height (DJ-H)”, and 2) “drop jump for maximum height and minimum ground CT (DJ-H/t)”. In that study, despite the lack of significant changes in jumping performance, the DJ-H group exhibited an increase of 6% in standing vertical jump height, against an increase of 3% in the DJ-H/t group. Nevertheless, the DJ-H/t program elicited greater improvements in reactive strength (+ 20%, against a 0.5% increase in the DJ-H group; determined by the best ratio between drop jump height and CT [cm•s^−1^]), which corroborates with the theory that this drop jump style can be more effective in maximizing rapid force generation and power-related capabilities (especially in the leg extensor muscles) (Bobbert et al., 1987; Pedley et al., 2017; Young et al., 1999). In a 12-week intervention analyzing the effects of two plyometric protocols in college athletes, both drop and countermovement jumps were shown to be effective at enhancing jumping ability (assessed by means of the squat, countermovement, and drop jump height), but the % changes for all jump types were greater in the drop jump group (Gehri et al., 1998). A key finding in that study was the lack of improvements in the utilization of stored elastic energy observed for both groups (calculated using methods described by Komi and Bosco (1978)), which also reinforces previous findings on the crucial importance of verbal cues in drop jump training (Bobbert et al., 1987; Khuu et al., 2015; Oliver et al., 2021; Pedley et al., 2017; Struzik et al., 2016). This is because both groups were instructed to rebound upward in a maximal vertical jump, without any additional instruction regarding ground CT. As a consequence, increases in vertical jump height were attributed to enhanced contractile performance in muscles involved in the jump tasks (and not to the use of stored elastic energy) (Gehri et al., 1998). A final point to consider when designing drop jump training programs is the adequate prescription of drop jump height. Although there is no consensus on the best dropping height to be applied during training, some authors have recommended the use of the “optimal drop height” (i.e., the height that elicits the highest ratio of jump height to CT; a variable often termed as “reactive strength index”) (Flanagan and Comyns, 2008; Jarvis et al., 2022; Newton and Dugan, 2002). A recent meta-analysis including 32 studies on this topic (Jarvis et al., 2022) identified moderate and large (negative and positive) associations between the reactive strength index and a number of independent measures of physical performance (*r* = 0.34 for pooled isometric and isotonic strength; *r* = −0.43 and −0.33 for sprint times across acceleration and top-speed phases; and *r* = −0.56 for change of direction speed times, respectively). Training studies with athletes from different sports and age-categories have compared the effects of using or not using the optimal drop height during pure plyometric or combined (plyometric + resistance) training, yielding controversial results (Ramirez-Campillo et al., 2018; Sotiropoulos et al., 2023). For example, an individualized drop jump training program (based on the optimal drop height) was superior to a “fixed drop-box height” program (i.e., using a 30-cm box) to develop a range of physical fitness attributes in a population of young soccer players (Ramirez-Campillo et al., 2018). In contrast, when combined with resistance training (i.e., half squat, leg extension, leg curl and some upper-body exercises), drop jump training performed from the optimal drop height or from a height 25% higher, resulted in greater increases in vertical jump height and the reactive strength index (compared to drop jump training performed from a drop height 25% lower than the optimal drop height, in highly-trained female volleyball players) (Sotiropoulos et al., 2023). Nonetheless, notably, training from a drop height 25% higher than the optimal drop height led to greater enhancements in various drop jump measures (i.e., increases in drop jump height and the reactive strength index, and decreases in ground CT). Therefore, for highly specialized athletes, the utilization of drop heights moderately higher (i.e., 25–30% higher) than the optimal drop height may be recommended to optimize the overall development of reactive strength and SSC efficiency. Still in this context, and specifically for highly-trained sprinters and rugby players, previous studies have demonstrated that, instead of the reactive strength index, the maximum height achieved after drop jumps executed from greater drop heights (i.e., ≥ 75 cm) is, in fact, the best predictor of sprinting speed over a range of distances (from 10- to 60-m) (Barr and Nolte, 2011; Loturco et al., 2019). This also supports the use of drop heights higher than the optimal drop height to train and assess elite athletes from different sports. However, practitioners should bear in mind that an elevated mechanical stress is also generated when using greater drop heights, which potentially increases the likelihood of acute and chronic injuries (Flanagan and Comyns, 2008; Peng, 2011; Sotiropoulos et al., 2023). In this regard, as abovementioned, the optimal drop height could also serve as a basis for the adequate selection and prescription of drop jump height, even when higher (or lower) drop heights are recommended (Flanagan and Comyns, 2008; Pedley et al., 2017; Sotiropoulos et al., 2023).

#### Box Jumps

**Figure 3:**
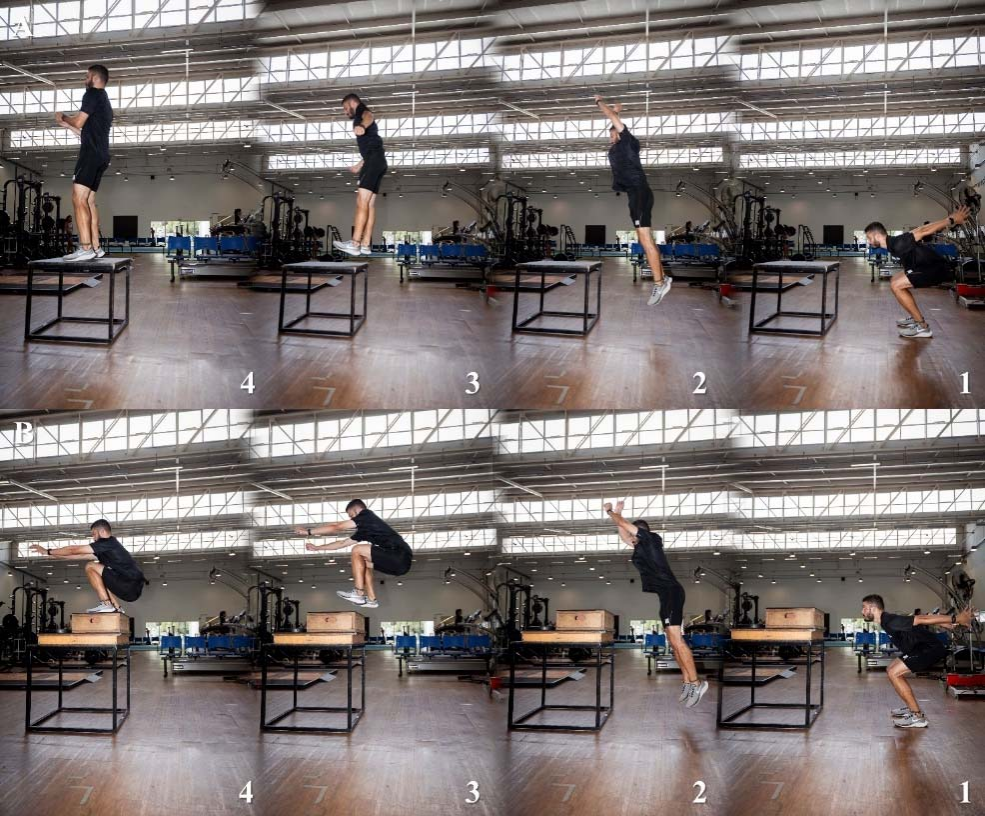
Box jumps execution, under two different conditions: “using the optimal box height” (Panel A) and “using an exaggerated box height” (Panel B), at the initial (1), flight (2), final approaching (3), and landing (4) phases.

Box jumps are widely used in several sports and among different age-categories (Fort-Vanmeerhaeghe et al., 2016; Keller et al., 2020; Michailidis et al., 2013; Waller et al., 2014, 2019), mainly because they allow the reduction in ground reaction forces when landing, thus limiting the increased risk of injury commonly associated with excessive eccentric loads (Booth and Orr, 2016; Koefoed et al., 2022; Prapavessis et al., 2003; Vescovi et al., 2008). For executing this exercise, athletes start from a standing position, in front of a plyometric box, before lowering their bodies, by flexing their hips, knees, and ankles (Koefoed et al., 2022). Athletes then jump explosively forward and upward, landing with both feet on top of the box (Figure 3). Although the intensity of box jumps is thought to be related to the box height, this is still a matter for debate. For Koefoed et al. (2022), this may be a “flawed assumption”, as the height of the box has a very limited influence on takeoff mechanics. In contrast, higher box heights place greater demands on flexibility and mobility (especially in hip and knee joints) at the landing phase, a movement pattern that is seldom observed in real sport actions (Koefoed et al., 2022; Waller et al., 2014). Higher boxes will also increase the risk of falling (Koefoed et al., 2022), a problem that may be even more critical for powerful athletes, who tend to jump higher and, hence, use high boxes during plyometric training sessions. From a rational perspective, this makes no sense, as jump intensity will be primarily driven by the intent to execute a maximal or a submaximal jump (Koefoed et al., 2022). Therefore, athletes can perform maximal jumps even when utilizing small or moderate box heights. In this regard, boxes will only serve as a landing area, with the intention of reducing eccentric load and dissipating the impulse at landing. For some authors, the ideal and safe height to perform box jumps should be calculated and defined on the basis of the athlete’s countermovement jump height (Koefoed et al., 2022; Waller et al., 2014). For example, plyometric boxes with a height ~20% lower than the maximal countermovement jump will ensure an adequate forward movement onto the box, avoiding athletes from tucking their knees up to their chest while in the air (Koefoed et al., 2022). Another criterion suggested for identifying the optimal box height may be based on the observation of knee flexion while landing on the top of the box. For Barnes (2003), a soft and safe landing can be achieved when the box height allows athletes to land “with their knees bent to approximately 120 degrees”. Due to their low-to-moderate intensity nature, box jumps are usually prescribed during preparatory or transitional training periods in order to develop and improve basic jumping abilities (Mothersole, 2013; Turner and Jeffreys, 2010; Waller et al., 2014, 2019) or in conjunction with more intense exercises (e.g., heavy squats, loaded squat jumps, and drop jumps) to increase a variety of neuromuscular qualities (e.g., the rate of force development, concentric force production, and power) across different phases of the season (as illustrated in Tables 1, 2, and 3) (Anthony and Baghurst, 2019; Dann and Kelly, 2021; Janz and Malone, 2008; Lockie et al., 2012; Marcello et al., 2017; Wing, 2018). Such a wide variety of applications combined with an inherent practicality and efficiency contribute to making the box jump one of the most prevalent jump drills not only within the sprint coaching community (Bolger et al., 2016; Healy et al., 2021), but among practitioners from different sports and of diverse performance levels, either to enhance the landing technique (and reduce landing forces) or improve athletic performance (Loturco et al., 2022, 2023b; McNeely, 2005; Mothersole, 2013; Weldon et al., 2022b).

#### Assisted Jumps

**Figure 4:**
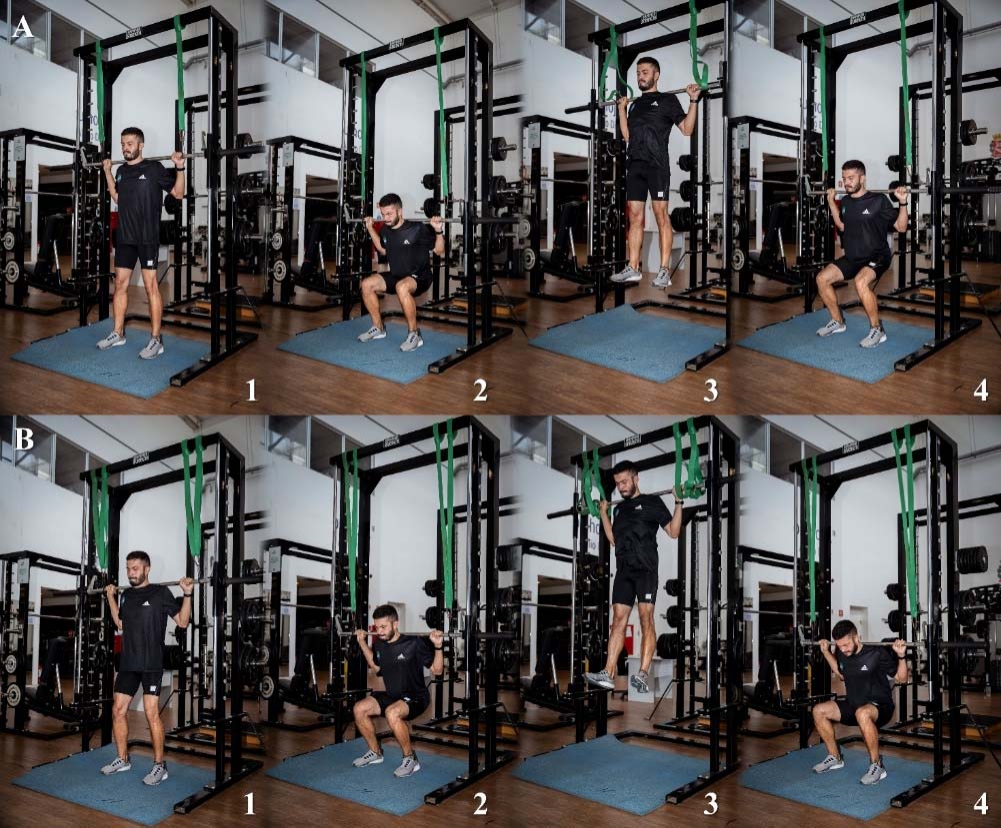
Assisted jumps execution, under two different conditions: “using the adequate elastic resistance” (Panel A) and “using an excessive elastic resistance” (Panel B), at the starting standing position (1), initial portion of the concentric action (2), and at the flight (3) and landing (4) phases.

Assisted jumps may be considered an overspeed training method, as this allows manipulation of the movement velocity by artificially increasing the maximum vertical acceleration rate while jumping, thus working as an additional training stimulus for speed and power development (Argus et al., 2011; Cazas et al., 2013; Loturco et al., 2015b, 2018). To increase movement velocity, practitioners usually utilize elastic bands with different resistance levels. The greater the assistance used (i.e., higher elastic resistances), the greater the jump-squat velocity and the resultant jump height (Figure 4) (Kilgallon and Beard, 2010; Loturco et al., 2015b). This alternative training method is ranked as the 6^th^ most popular form of jump exercise in the preference list of Brazilian Olympic sprint and jump coaches, being considered a very effective and safe way to develop lower limb power in elite junior track and field and team sport athletes (Kilgallon and Beard, 2010; Loturco et al., 2015b, 2018). In a more applied perspective, by unloading the athletes’ body-mass with the aid of elastic bands, it is possible to reduce mechanical stress imposed by the traditional loading approach on the back, and on the knee and hip joints, making this strategy particularly interesting for younger athletes (Kilgallon and Beard, 2010; Loturco et al., 2015b). In addition to this, it has been advocated that higher velocities achieved during assisted jumps can potentially lead to positive adaptations in the high-velocity portion of the force-velocity spectrum, which is consistent with previous studies on this topic (Cazas et al., 2013; Cook et al., 2013; Kilgallon and Beard, 2010; Loturco et al., 2015b; Markovic et al., 2011; Sheppard et al., 2011). Collectively, the empirical evidence regarding the effectiveness of assisted jumps on power production at unloaded and very-light loading conditions and sprint speed (Cazas et al., 2013; Cook et al., 2013; Kilgallon and Beard, 2010; Loturco et al., 2015b; Markovic et al., 2011; Sheppard et al., 2011), could explain, for example, their large utilization by Olympic sprint coaches during both preparatory and competitive periods (Table 1) (Loturco et al., 2023b). Another important aspect to be considered when prescribing negative loads is the proper selection of band types. Although bands with greater resistances can be used to elicit faster movement velocities, they can also lead to excessive jump heights and, as a consequence, increase the likelihood of jump-landing injuries (Kilgallon and Beard, 2010). A good strategy for selecting adequate bands may be the utilization of movement velocity as a reference variable for determining the level of resistance for assisted jumps. Loturco et al. (2015b) proposed an increment of 20% in jump squat velocity (as compared with the “velocity of reference”; namely, the bar-velocity assessed during jump squats without any additional load placed on the barbell) for improving speed and power performance in elite under-20 soccer players. Using this criterion, it is possible to preserve the biomechanical characteristics of the ballistic exercise, as reduced and well-balanced variations in jump squat velocity still allow athletes to maintain good levels of coordination and technique (e.g., movement pattern, balance, and posture) and apply considerable amounts of force while jumping. Moreover, by adjusting the intensity of assisted jumps with the use of the velocity-based approach, athletes with distinct characteristics (e.g., heavier, and lighter subjects, or individuals with different levels of relative strength) will execute the exercise at a similar loading intensity (e.g., 20% faster than the traditional unloaded condition, as defined by the velocity of reference). In contrast, strategies based on fixed percentages of body-mass reduction (e.g., −30% of body-mass) may compromise the monitoring and prescription of training intensity, which can be even more problematic in team-sports, where athletes with very different anthropometric traits and physical performance profiles (e.g., rugby backs and forwards, basketball point guards and power forwards) will probably experience different loading intensities under similar percentages of body-mass reduction (Loturco et al., 2015b). In summary, despite their proven effectiveness, great popularity, and perceived ease of use, assisted jumps should be properly prescribed and controlled, in order to enhance their potential benefits and reduce safety concerns. The correct selection of elastic bands is, undoubtedly, the most important factor in this process.

#### Bounding

**Figure 5:**
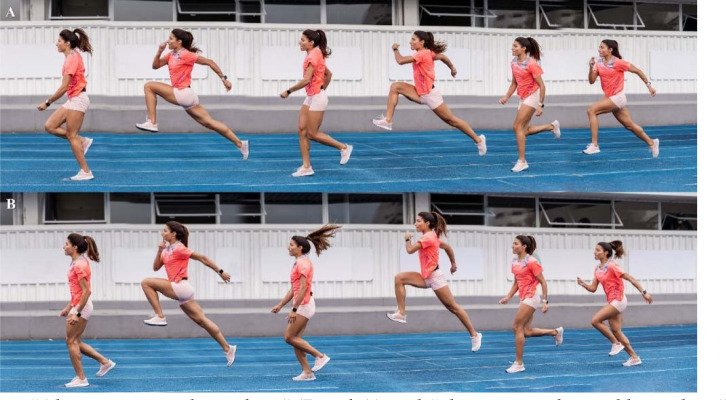
“Alternate sprint bounding” (Panel A) and “alternate traditional bounding” sequencing (Panel B).

Bounding is a type of “multiple jumping exercise for maximum distance” (Young, 1992) that can be performed in single, double, and alternate jump modes. This horizontally-oriented drill is usually executed with the main objective of improving the ability to apply force in the horizontal direction and, thus, mechanical efficiency during the acceleration phase of sprint running (Behrens and Simonson, 2011; Young, 1992). Indeed, a recent meta-analysis confirmed the superiority of horizontally-oriented jumps (compared to vertically-oriented jumps) at improving horizontal performance (Moran et al., 2021), which may explain the extensive use of this type of exercise among Brazilian Olympic sprint and jump coaches (who ranked bounding as the 2^nd^ most commonly prescribed exercise out of eight different plyometric activities) (Loturco et al., 2023b). Due to its inherent characteristics (i.e., exaggerated running strides while swinging arms) (Chu, 1998) and similarity with sprinting technique (Turner et al., 2015), “alternate bounding” is the most commonly researched and used mode of bounding in a wide variety of sport disciplines (Figure 5) (Chu, 1985; Dann et al., 2023; Turner et al., 2015; Young, 1992). Overall, there are two forms of alternate bounding, which may be defined and selected according to their mechanical aspects: “traditional bounding” and “sprint bounding”. For both exercises, the transition between the eccentric action, initiated at the beginning of the contact phase, and the concentric action (i.e., muscle shortening over the push-off phase) occurs unilaterally (i.e., one-foot take-off) and horizontally, involving an alternate running arm swing (Chu, 1985; Young, 1992). The main difference between these two respective bounding forms is related to the time spent during the ground contact phase and, consequently, to the changes in neuromuscular activity and movement patterns that arise from this observation. While traditional bounding involves landing on the heels and CT of ~200 ms, sprint bounding results in “more active landings” (i.e., flat-foot contact), with shorter CT (~130 ms) (Young, 1992). Therefore, although both exercises can be used to improve acceleration capacity, sprint bounding seems to be more effective to develop maximum speed, as this better reflects (or at least approaches) the actual demands and CTs recorded during top-speed phases (Young, 1992) (i.e., CT < 100 ms, for elite sprinters) (Mattes et al., 2021; Mero et al., 1992). Despite its similarity to sprinting technique and horizontal dominance, the transference of bounding to sprint performance appears to be optimized when this plyometric drill is combined with other jump types (e.g., hurdle, drop, and tuck jumps) and/or training modes (e.g., resistance training) (Duvall, 1993; Moran et al., 2021; Rimmer and Sleivert, 2000; Young et al., 2001). As sprinting is a complex skill, most authors who investigate this topic propose the use of mixed training strategies for enhancing sprint performance, including not only bounding drills or other horizontally-oriented techniques (e.g., sled towing), but also a variety of vertically-oriented jumps and strength-power exercises (e.g., squats and Olympic lifts), which may be balanced and prescribed according to athletes’ needs and objectives (Haugen et al., 2019; Loturco et al., 2023b; Young, 2001, 2006). A final and crucial point to consider is that, irrespective of athletes’ characteristics, plyometric programs designed to improve sprint speed (especially over short-distances) and horizontal force production should have a predominance of horizontally-oriented jumps (over vertically-oriented jumps) in their training sessions (Moran et al., 2021). Under this perspective, the use of bounding jumps, in all their different modes (i.e., single, double, and alternate modes) and forms (i.e., traditional and sprint bounding) is highly recommended.

#### Standing Long Jumps and Multiple Hops

**Figure 6:**
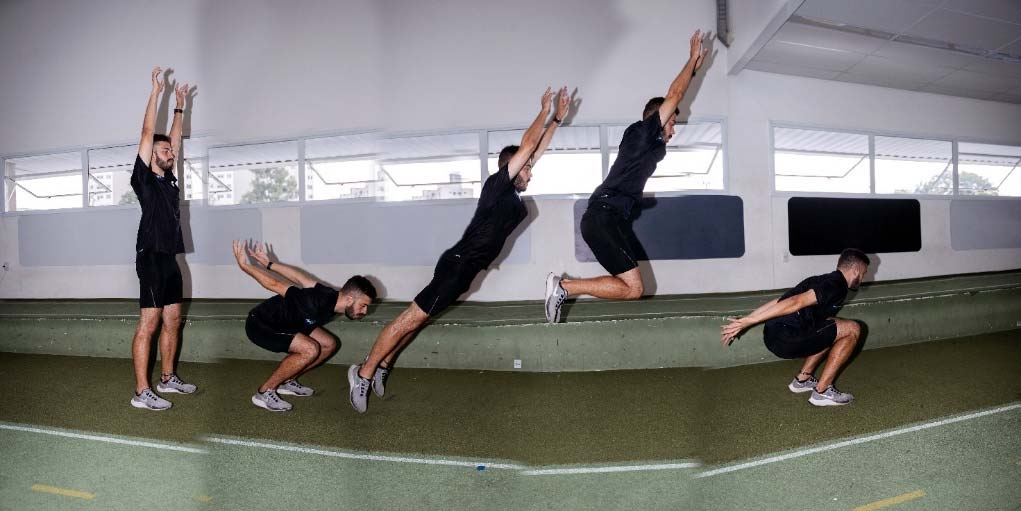
Standing long jump sequencing.

**Figure 7:**
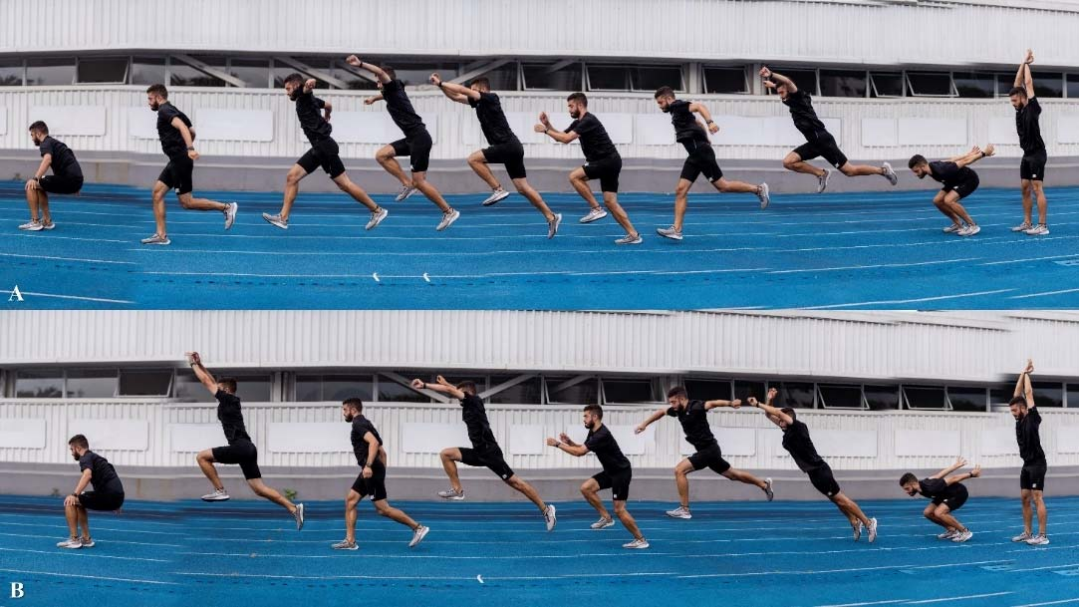
Multiple hops sequencing: “single-leg stance multiple hop” performed on the right leg (Panel A) and “double-leg stance multiple hop” (Panel B).

Standing long jumps and multiple hops are variations of horizontally-oriented drills which share a common particularity: both of them are usually performed from a static standing posture (Figures 6 and 7). As a result, these jumps may be more appropriate for improving (and indirectly assessing) maximum acceleration capacity (Hudgins et al., 2013; Loturco et al., 2015a; Moresi et al., 2011; Sole, 2018), especially in situations where athletes have to sprint as fast as possible from stationary positions, such as, for example, during block (or static) starts in track and field events. These potential applications are particularly stimulated by a series of studies, which demonstrated the close relationships that exist between standing long jump (executed either individually or sequentially) and multiple hop performances and the sprint acceleration phase, a fact that is even more pronounced in elite sprinters and jumpers (Habibi et al., 2010; Loturco et al., 2015a; Maćkała et al., 2015; Maulder and Cronin, 2005). Another common characteristic of these jumps is that both can be performed in single- or double-leg stances which, along with their simplicity, easiness of implementation, and cost-effectiveness (i.e., jump distance may be determined using a simple metric tape) make these exercises very popular as alternative and indirect forms of estimating lower-limb power in both young and adult athletic populations (Fernandez-Santos et al., 2015; Hudgins et al., 2013; Pullen et al., 2022). When performed sequentially, standing long jumps and multiple hops are normally prescribed and tested within pre-determined and fixed constraints (e.g., triple, quintuple or decuple jumps) (Aoki et al., 2015; Hudgins et al., 2013; Pereira et al., 2016), thereby facilitating the collection and establishment of normative data for these measurements in different sports, competitive levels, and age categories (Coburn, 2012; Martinez-de-Quel et al., 2021; Selmi et al., 2020). Despite these similarities, the frequency of use of standing jumps and multiple hops among Brazilian Olympic sprint and jump coaches is very different: whereas 68% of them declared regularly prescribing multiple hops, only 21% of these coaches reported frequently using standing jumps in their training programs (Loturco et al., 2023b). Possible explanations for these discrepancies include the primary utilization of static slower jumps during the initial phases of athletes’ preparation, either as assessment routines or as a form of basic (foundation) training, aspects that may compromise or at least reduce their use during competitive training periods. On the other hand, jump coaches may also continue to prescribe multiple hops from static positions to artificially increase ground CT and the duration of braking and propulsion (i.e., push-off) phases, in an attempt to reproduce the biomechanics of the triple jump (as horizontal speed decreases and CT gradually increases from the hop to the jump) (Perttunen et al., 2000). Among other things, this may explain why multiple hops are more regularly used than standing long jumps over the entire training season (Loturco et al., 2023b). Generally speaking, horizontally-oriented static drills, such as standing long jumps and multiple hops, executed in single- or double-leg stances, can be effectively used to enhance acceleration capacity in the initial stages of plyometric training and to indirectly assess lower-limb power in athletes from a range of sports (Brechue et al., 2010; Hudgins et al., 2013; Weldon et al., 2022b). Athletes interested in improving their ability to apply greater amounts of force at slower horizontal speeds and during longer ground CTs (e.g., triple jumpers and athletes with a greater need for acceleration improvements) should also include these exercises as part of their regular training sessions.

## Conclusions and Practical Implications

Hurdle, drop, box, and assisted jumps, along with bounding, standing long jumps, and multiple hops are the most commonly used plyometric exercises by Brazilian Olympic sprint and jump coaches. The extensive experience and knowledge of these coaches may help practitioners from different sport disciplines to better design plyometric training programs. In general, plyometric sessions are prescribed across the entire training season, with higher frequency during the preparatory period (compared to the competitive period; 2–3 vs. 1–2 times per week, respectively). Hurdle jumps are the most popular exercises, as they more closely resemble the biomechanical characteristics of sprint and jump events (and many sport-specific tasks), thus yielding good transference to performance. Drop jumps are prescribed with different aims (i.e., improving sprint speed, jumping ability, running economy), using the RSI as a reference measure to determine dropping height. Verbal cues have a key role in mediating responses to drop jump training: athletes with the intention of increasing jump height should be instructed to maximize jump height; athletes with the intention of improving reactive strength should be instructed to jump as high and as quickly as possible. Box jumps are primarily used to reduce ground reaction forces while landing and should be performed using boxes regulated by the individual’s CMJ height. For box jumps, higher boxes do not represent higher intensities and may even increase the risk of falling. In this exercise, the intensity is determined by the intention to execute the movement as forcefully and as powerfully as possible. Assisted jumps may be used as an overspeed training method, specifically to improve the ability to produce force at high- and very-high velocities. The correct selection of elastic bands is essential for the adequate and safe prescription of negative loads. Bounding is a multiple jumping horizontally-oriented drill executed with the main objective of increasing horizontal force production and, hence, sprinting speed. Sprint bounding may be more effective than “traditional bounding drills” in this regard. Standing long jumps and multiple hops may also be classified as horizontal drills; however, these jumps are performed at slower speeds, from static (stationary) positions. Therefore, they can be more appropriate for developing maximum acceleration capacity or the ability to apply greater amounts of force during longer CTs. In addition, these static jumps may be used as indirect measurements of lower-limb power, either to assess or to classify athletes from various sports, performance levels, and age categories. Overall, all these exercises have already been proven to be effective in improving a range of speed and power qualities, and may be used alone, combined among themselves, or in combination with other training methods (e.g., plyometrics + resistance training). The rational used by Olympic sprint and jump coaches along with the practical information discussed in this article may serve as a relevant guide for coaches of different sports and levels.

## References

[ref1] Anthony, C. C., & Baghurst, T. M. (2019). Strength and conditioning considerations for racquetball athletes. Strength and Conditioning Journal, 41(3), 24–34.

[ref2] Aoki, K., Kohmura, Y., Sakuma, K., Koshikawa, K., & Naito, H. (2015). Relationships between field tests of power and athletic performance in track and field athletes specializing in power events. International Journal of Sports Science and Coaching, 10(1), 133–144.

[ref3] Áragón-Vargas, L. F., & Gross, M. M. (1997). Kinesiological factors in vertical jump performance: Differences within individuals. Journal of Applied Biomechanics, 13(1), 45–65.

[ref4] Argus, C. K., Gill, N. D., Keogh, J. W., Blazevich, A. J., & Hopkins, W. G. (2011). Kinetic and training comparisons between assisted, resisted, and free countermovement jumps. Jorunal of Strength and Condiotinig Research, 25(8), 2219–2227. 10.1519/JSC.0b013e3181f6b0f4 21654341

[ref5] Barker, L., Bankers, S., Farmer, B., Siedlik, J., Harry, J., & Grindstaff, T. L. (2021). The Influence of Verbal Cues on Drop Jump Landing Strategies in NCAA Division I Soccer Players. American Journal of Sports Science, 9(2), 37–42.

[ref6] Barnes, M. (2003). Introduction to Plyometrics. NSCAs Performance Training Jorunal, 2(2), 13–20.

[ref7] Barr, M. J., & Nolte, V. W. (2011). Which measure of drop jump performance best predicts sprinting speed? Jorunal of Strength and Conditioning Research, 25(7), 1976–1982. 10.1519/JSC.0b013e3181e4f7ba 21701285

[ref8] Behrens, M. J., & Simonson, S. R. (2011). A comparison of the various methods used to enhance sprint speed. Strength and Conditioning Journal, 33(2), 64–71.

[ref9] Bobbert, M. F. (1990). Drop jumping as a training method for jumping ability. Sports Medicine, 9(1), 7–22. 2408119 10.2165/00007256-199009010-00002

[ref10] Bobbert, M. F., Huijing, P. A., & van Ingen Schenau, G. J. (1987). Drop jumping. I. The influence of jumping technique on the biomechanics of jumping. Medicine and Science in Sports and Exercise, 19(4), 332–338. 3657481

[ref11] Bolger, R., Lyons, M., Harrison, A. J., & Kenny, I. C. (2016). Coaching sprinting: Expert coaches’ perception of resistance-based training. International Journal of Sports Science and Coaching, 11(5), 746–754.

[ref12] Booth, M. A., & Orr, R. (2016). Effects of plyometric training on sports performance. Strength and Conditioning Journal, 38(1), 30–37.

[ref13] Boullosa, D. (2021). Post-activation performance enhancement strategies in sport: a brief review for practitioners. Human Movement, 22(3), 101–109.

[ref14] Brechue, W. F., Mayhew, J. L., & Piper, F. C. (2010). Characteristics of sprint performance in college football players. Journal of Strength and Conditioning Research, 24(5), 1169–1178. 20386124 10.1519/JSC.0b013e3181d68107

[ref15] Canata, G. L., & Casale, V. (2022). Athletics: Jumping. In G. L. Canata., & H., Jones (Eds.), Epidemiology of Injuries in Sports. Berlin: Springer.

[ref16] Cappa, D. F., & Behm, D. G. (2011). Training specificity of hurdle vs. countermovement jump training. Journal of Strength and Conditioning Research, 25(10), 2715–2720. 21873903 10.1519/JSC.0b013e318208d43c

[ref17] Cappa, D. F., & Behm, D. G. (2013). Neuromuscular characteristics of drop and hurdle jumps with different types of landings. Journal of Strength and Conditioning Research, 27(11), 3011–3020. 23442288 10.1519/JSC.0b013e31828c28b3

[ref18] Cazas, V. L., Brown, L. E., Coburn, J. W., Galpin, A. J., Tufano, J. J., Laporta, J. W., & Du Bois, A. M. (2013). Influence of rest intervals after assisted jumping on bodyweight vertical jump performance. Journal of Strength and Conditioning Research, 27(1), 64–68. 23085973 10.1519/JSC.0b013e3182772f13

[ref19] Chelly, M. S., Ghenem, M. A., Abid, K., Hermassi, S., Tabka, Z., & Shephard, R. J. (2010). Effects of in-season short-term plyometric training program on leg power, jump-and sprint performance of soccer players. Journal of Strength and Conditioning Research, 24(10), 2670–2676. 20844458 10.1519/JSC.0b013e3181e2728f

[ref20] Chelly, M. S., Hermassi, S., & Shephard, R. J. (2015). Effects of In-Season Short-term Plyometric Training Program on Sprint and Jump Performance of Young Male Track Athletes. Journal of Strength and Conditioning Research, 29(8), 2128–2136. 25647644 10.1519/JSC.0000000000000860

[ref21] Chu, D. (1986). Practical considerations for utilizing plyometrics. National Strength and Conditioning Association Journal, 8(3), 14–22.

[ref22] Chu, D. A. (1985). Jumping into Plyometrics: Alternate bounding. Strength and Conditioning Journal, 7(2), 59–59.

[ref23] Chu, D. A. (1998). *Jumping into plyometrics*. Champaign, IL: Human Kinetics.

[ref24] Clark, K. (2018). The need for speed-improving sprinting performance in football players. National Strength and Conditioning Association Coach, 5(4), 14–22.

[ref25] Coburn, J. W. (2012). Measuring power. Strength and Conditioning Journal, 34(6), 25–28.

[ref26] Comfort, P., & Matthews, M. (2010). Strength and conditioning. In P., Comfort & E., Abrahamson (Eds.), Sports rehabilitation and injury prevention (pp. 233–244). Chichester, UK: Wiley-Blackwell.

[ref27] Cook, C. J., Beaven, C. M., & Kilduff, L. P. (2013). Three weeks of eccentric training combined with overspeed exercises enhances power and running speed performance gains in trained athletes. Journal of Strength and Conditioning Research, 27(5), 1280–1286. 22820207 10.1519/JSC.0b013e3182679278

[ref28] Cormier, P., Freitas, T. T., Loturco, I., Turner, A., Virgile, A., Haff, G. G., Bishop, C. (2022). Within Session Exercise Sequencing During Programming for Complex Training: Historical Perspectives, Terminology, and Training Considerations. Sports Medicine, 52(10), 2371–2389. 35816233 10.1007/s40279-022-01715-x

[ref29] Dann, E., Quinn, S., Russell, M., Kilduff, L. P., Turner, A. N., & Hills, S. P. (2023). Alternate Leg Bounding Acutely Improves Change of Direction Performance in Women's Team Sports Players Irrespective of Ground Type. Journal of Stregnth and Conditioning Research, 37(6), 1199–1203. 10.1519/JSC.0000000000004378 36394564

[ref30] Dann, R. A., & Kelly, V. G. (2021). Evidence-based strength and conditioning plan for freestyle snowboarding athletes. Strength and Conditioning Journal, 43(5), 1–11.

[ref31] Dintiman, G. B., & Ward, R. D. (1988). *Sport speed*. Champaign, IL: Leisure Press.

[ref32] Douglas, J., Pearson, S., Ross, A., & McGuigan, M. (2018). Kinetic Determinants of Reactive Strength in Highly Trained Sprint Athletes. Journal of Strength and Conditioning Research, 32(6), 1562–1570. 28930875 10.1519/JSC.0000000000002245

[ref33] Duvall, M. A. (1993). Explosive Training: Implementing Plyometrics in an In-Season Football Program. Strength and Conditioning Journal, 15(3), 57–59.

[ref34] Fatouros, I. G., Jamurtas, A. Z., Leontsini, D., Taxildaris, K., Aggelousis, N., Kostopoulos, N., & Buckenmeyer, P. (2000). Evaluation of plyometric exercise training, weight training, and their combination on vertical jumping performance and leg strength. Journal of Strength and Conditioning Research, 14(4), 470–476.

[ref35] Fernandez-Santos, J. R., Ruiz, J. R., Cohen, D. D., Gonzalez-Montesinos, J. L., & Castro-Piñero, J. (2015). Reliability and Validity of Tests to Assess Lower-Body Muscular Power in Children. Journal of Strength and Conditioning Research, 29(8), 2277–2285. 25647647 10.1519/JSC.0000000000000864

[ref36] Flanagan, E. P., & Comyns, T. M. (2008). The use of contact time and the reactive strength index to optimize fast stretch-shortening cycle training. Strength and Conditioning Journal, 30(5), 32–38.

[ref37] Fort-Vanmeerhaeghe, A., Romero-Rodriguez, D., Lloyd, R. S., Kushner, A., & Myer, G. D. (2016). Integrative neuromuscular training in youth athletes. Part II: Strategies to prevent injuries and improve performance. Strength and Conditioning Journal, 38(4), 9–27.

[ref38] Fouré, A., Nordez, A., & Cornu, C. (2012). Effects of plyometric training on passive stiffness of gastrocnemii muscles and Achilles tendon. European Journal of Applied Physiology, 112(8), 2849–2857. 22131086 10.1007/s00421-011-2256-x

[ref39] Fukashiro, S., Hay, D. C., & Nagano, A. (2006). Biomechanical behavior of muscle-tendon complex during dynamic human movements. Journal of Applied Biomechanics, 22(2), 131–147. 16871004 10.1123/jab.22.2.131

[ref40] Gehri, D. J., Ricard, M. D., Kleiner, D. M., & Kirkendall, D. T. (1998). A comparison of plyometric training techniques for improving vertical jump ability and energy production. Journal of Strength and Conditioning Research, 12(2), 85–89.

[ref41] Habibi, A., Shabani, M., Rahimi, E., Fatemi, R., Najafi, A., Analoei, H., & Hosseini, M. 2010. Relationship between jump test results and acceleration phase of sprint performance in national and regional 100m sprinters. Journal of Human Kinetics, 23, 29–35.

[ref42] Harrison, P. W., James, L. P., McGuigan, M. R., Jenkins, D. G., & Kelly, V. G. (2019). Resistance Priming to Enhance Neuromuscular Performance in Sport: Evidence, Potential Mechanisms and Directions for Future Research. Sports Medicine, 49(10), 1499–1514. 31203499 10.1007/s40279-019-01136-3

[ref43] Haugen, T., Seiler, S., Sandbakk, Ø., & Tønnessen, E. (2019). The Training and Development of Elite Sprint Performance: an Integration of Scientific and Best Practice Literature. Sports Medicine Open, 5(1), 44. 31754845 10.1186/s40798-019-0221-0 PMC6872694

[ref44] Healy, R., Bolger, R., Kenny, I. C., & Harrison, A. J. (2020). Kinematic differences between sprinting and the hurdle jump exercise–a preliminary analysis. ISBS Proceedings Archives, 38(1), 520.

[ref45] Healy, R., Kenny, I. C., & Harrison, A. J. (2021). Resistance Training Practices of Sprint Coaches. Journal of Strength and Conditioning Research, 35(7), 1939–1948. 30747902 10.1519/JSC.0000000000002992

[ref46] Hermassi, S., Chelly, M. S., Fathloun, M., & Shephard, R. J. (2010). The effect of heavy-vs. moderate-load training on the development of strength, power, and throwing ball velocity in male handball players. Journal of Strength and Conditioning Research, 24(9), 2408–2418. 20706155 10.1519/JSC.0b013e3181e58d7c

[ref47] Hudgins, B., Scharfenberg, J., Triplett, N. T., & McBride, J. M. (2013). Relationship between jumping ability and running performance in events of varying distance. Journal of Strength and Conditioning Research, 27(3), 563–567. 23222079 10.1519/JSC.0b013e31827e136f

[ref48] Husbands, C. (2013). *Sprinting: Training, Techniques and Improving Performance*. Ramsbury, UK: Crowood.

[ref49] Janz, J., & Malone, M. (2008). Training explosiveness: Weightlifting and beyond. Strength and Conditioning Journal, 30(6), 14–22.

[ref50] Jarvis, P., Turner, A., Read, P., & Bishop, C. (2022). Reactive Strength Index and its Associations with Measures of Physical and Sports Performance: A Systematic Review with Meta-Analysis. Sports Medicine, 52(2), 301–330. 34606061 10.1007/s40279-021-01566-y

[ref51] Keller, S., Koob, A., Corak, D., von Schöning, V., & Born, D. P. (2020). How to Improve Change-of-Direction Speed in Junior Team Sport Athletes-Horizontal, Vertical, Maximal, or Explosive Strength Training? Journal of Strength and Conditioning Research, 34(2), 473–482. 30199451 10.1519/JSC.0000000000002814

[ref52] Khuu, S., Musalem, L. L., & Beach, T. A. (2015). Verbal Instructions Acutely Affect Drop Vertical Jump Biomechanics--Implications for Athletic Performance and Injury Risk Assessments. Journal of Strength and Conditioning Research, 29(10), 2816–2826. 26398699 10.1519/JSC.0000000000000938

[ref53] Kilgallon, M., & Beard, A. (2010). The assisted jump squat: an alternative method for developing power in adolescent athletes. Strength and Conditioning Journal, 32(4), 26–29.

[ref54] Koefoed, N., Dam, S., & Kersting, U. G. (2022). Effect of Box Height on Box Jump Performance in Elite Female Handball Players. Journal of Strength and Conditioning Research, 36(2), 508–512. 32187147 10.1519/JSC.0000000000003481

[ref55] Komi, P. V., & Bosco, C. (1978). Utilization of stored elastic energy in leg extensor muscles by men and women. Medicine and Science in Sports, 10(4), 261–265. 750844

[ref56] Lees, A., Vanrenterghem, J., & De Clercq, D. (2004). The maximal and submaximal vertical jump: implications for strength and conditioning. Journal of Strength and Conditioning Research, 18(4), 787–791. 15574084 10.1519/14093.1

[ref57] Li, F., Newton, R. U., Shi, Y., Sutton, D., & Ding, H. (2021). Correlation of Eccentric Strength, Reactive Strength, and Leg Stiffness With Running Economy in Well-Trained Distance Runners. Journal of Strength and Conditioning Research, 35(6), 1491–1499. 31809458 10.1519/JSC.0000000000003446

[ref58] Lockie, R. G., Murphy, A. J., Schultz, A. B., Knight, T. J., & Janse de Jonge, X. A. (2012). The effects of different speed training protocols on sprint acceleration kinematics and muscle strength and power in field sport athletes. Journal of Strength and Conditioning Research, 26(6), 1539–1550. 21912294 10.1519/JSC.0b013e318234e8a0

[ref59] Loturco, I., D'Angelo, R. A., Fernandes, V., Gil, S., Kobal, R., Cal Abad, C. C., Nakamura, F. Y. (2015a). Relationship between sprint ability and loaded/unloaded jump tests in elite sprinters. Journal of Strength and Conditioning Research, 29(3), 758–764. 25162648 10.1519/JSC.0000000000000660

[ref60] Loturco, I., Fernandes, V., Bishop, C., Mercer, V. P., Siqueira, F., Nakaya, K., Haugen, T. (2023a). Variations in Physical and Competitive Performance of Highly Trained Sprinters Across an Annual Training Season. Journal of Strength and Conditioning Research, 37(5), 1104–1110 36730012 10.1519/JSC.0000000000004380

[ref61] Loturco, I., Freitas, T. T., Alcaraz, P. E., Kobal, R., Hartmann Nunes, R. F., Weldon, A., & Pereira, L. A. (2022). Practices of strength and conditioning coaches in Brazilian elite soccer. Biology of Sport, 39(3), 779–791. 35959323 10.5114/biolsport.2022.108703 PMC9331335

[ref62] Loturco, I., Haugen, T., Freitas, T. T., Bishop, C., Moura, T. B. M. A., Mercer, V. P., Weldon, A. (2023b). Strength and conditioning practices of Brazilian Olympic sprint and jump coaches. Journal of Human Kinetics, 86(1), 175–194. 37181261 10.5114/jhk/159646 PMC10170547

[ref63] Loturco, I., Kobal, R., Kitamura, K., Fernandes, V., Moura, N., Siqueira, F., Pereira, L. A. (2019). Predictive Factors of Elite Sprint Performance: Influences of Muscle Mechanical Properties and Functional Parameters. Journal of Strength and Conditioning Research, 33(4), 974–986. 30913203 10.1519/JSC.0000000000002196

[ref64] Loturco, I., McGuigan, M. R., Freitas, T. T., Valenzuela, P. L., Pereira, L. A., & Pareja-Blanco, F. (2021). Performance and reference data in the jump squat at different relative loads in elite sprinters, rugby players, and soccer players. Biology of Sport, 38(2), 219–227. 34079166 10.5114/biolsport.2020.98452 PMC8139350

[ref65] Loturco, I., Nakamura, F. Y., Kobal, R., Gil, S., Abad, C. C., Cuniyochi, R., Roschel, H. (2015b). Training for Power and Speed: Effects of Increasing or Decreasing Jump Squat Velocity in Elite Young Soccer Players. Journal of Strength and Conditioning Research, 29(10), 2771–2779. 25807028 10.1519/JSC.0000000000000951

[ref66] Loturco, I., Pereira, L. A., Kobal, R., & Nakamura, F. Y. (2018). Using loaded and unloaded jumps to increase speed and power performance in elite young and senior soccer players. Strength and Conditioning Journal, 40(3), 95–103.

[ref67] Loturco, I., Pereira, L. A., Kobal, R., Zanetti, V., Kitamura, K., Abad, C. C., & Nakamura, F. Y. (2015c). Transference effect of vertical and horizontal plyometrics on sprint performance of high-level U-20 soccer players. Journal of Sports Science, 33(20), 2182–2191. 10.1080/02640414.2015.1081394 26390150

[ref68] Maćkała, K., Fostiak, M., & Kowalski, K. (2015). Selected determinants of acceleration in the 100m sprint. Journal of Human Kinetics, 45, 135–148. 25964817 10.1515/hukin-2015-0014 PMC4415826

[ref69] Makaruk, H., Starzak, M., Suchecki, B., Czaplicki, M., & Stojiljković, N. (2020). The Effects of Assisted and Resisted Plyometric Training Programs on Vertical Jump Performance in Adults: A Systematic Review and Meta-Analysis. Journa of Sports Science and Medicine, 19(2), 347–357. PMC7196747 32390728

[ref70] Marcello, R. T., Greer, B. K., & Greer, A. E. (2017). Acute Effects of Plyometric and Resistance Training on Running Economy in Trained Runners. Journal of Strength and Conditioning Research, 31(9), 2432–2437. 27806012 10.1519/JSC.0000000000001705

[ref71] Markovic, G. (2007). Does plyometric training improve vertical jump height? A meta-analytical review. British Journal of Sports Medicine, 41(6), 349–355. 17347316 10.1136/bjsm.2007.035113 PMC2465309

[ref72] Markovic, G., Vuk, S., & Jaric, S. (2011). Effects of jump training with negative versus positive loading on jumping mechanics. International Journal of Sports Medicine, 32(5), 365–372. 21380966 10.1055/s-0031-1271678

[ref73] Marshall, B. M., & Moran, K. A. (2013). Which drop jump technique is most effective at enhancing countermovement jump ability, “countermovement” drop jump or “bounce” drop jump? Journal of Sports Science, 31(12), 1368–1374. 10.1080/02640414.2013.789921 23631690

[ref74] Martinez-de-Quel, O., Alegre, L. M., Castillo-García, A., & Ayán, C. (2021). Anthropometric and fitness normative values for young karatekas. Biology of Sport, 38(3), 351–357. 34475618 10.5114/biolsport.2021.99324 PMC8329972

[ref75] Mattes, K., Wolff, S., & Alizadeh, S. (2021). Kinematic Stride Characteristics of Maximal Sprint Running of Elite Sprinters-Verification of the “Swing-Pull Technique”. Journal of Human Kinetics, 77, 15–24. 34168688 10.2478/hukin-2021-0008 PMC8008308

[ref76] Maulder, P., & Cronin, J. B. (2005). Horizontal and vertical jump assessment: reliability, symmetry, discriminative and predictive ability. Physical Therapy in Sport, 6(2), 74–82.

[ref77] McGuigan, M. R., Doyle, T. L., Newton, M., Edwards, D. J., Nimphius, S., & Newton, R. U. (2006). Eccentric utilization ratio: effect of sport and phase of training. Journal of Strength and Conditioning Research, 20(4), 992–995. 17194252 10.1519/R-19165.1

[ref78] McMahon, J. J. (2018). Stretch-shortening cycle and muscle-tendon stiffness. In A. Turner & P. Comfort (Eds.), Advanced Strength and Conditioning: An Evidence-based Approach (pp. 39–55). Abingdon, NY: Routledge.

[ref79] McNeely, E. (2005). Introduction to plyometrics: Converting strength to power. NSCAs Performance Training Journal, 6(5), 19–22.

[ref80] Mero, A., Komi, P. V., & Gregor, R. J. (1992). Biomechanics of sprint running. A review. Sports Medicine, 13(6), 376–392. 1615256 10.2165/00007256-199213060-00002

[ref81] Michailidis, Y., Fatouros, I. G., Primpa, E., Michailidis, C., Avloniti, A., Chatzinikolaou, A., Kambas, A. (2013). Plyometrics' trainability in preadolescent soccer athletes. Journal of Strength and Conditioning Research, 27(1), 38–49. 22450257 10.1519/JSC.0b013e3182541ec6

[ref82] Moran, J., Ramirez-Campillo, R., Liew, B., Chaabene, H., Behm, D. G., Garcia-Hermoso, A., Granacher, U. (2021). Effects of Vertically and Horizontally Orientated Plyometric Training on Physical Performance: A Meta-analytical Comparison. Sports Medicine, 51(1), 65–79. 32897526 10.1007/s40279-020-01340-6

[ref83] Moresi, M. P., Bradshaw, E. J., Greene, D., & Naughton, G. (2011). The assessment of adolescent female athletes using standing and reactive long jumps. Sports Biomechanics, 10(2), 73–84. 21834392 10.1080/14763141.2011.569564

[ref84] Mothersole, G. (2013). Ground reaction force profiles of specific jump-landing tasks in females: development of a systematic and progressive jump landing model. (Master's Thesis). Auckland University of Technology, Auckland, New Zealand. https://orapp.aut.ac.nz/handle/10292/5695 (accessed on 22 February 2023)

[ref85] Newton, R. U., & Dugan, E. (2002). Application of strength diagnosis. Strength and Conditioning Journal, 24(5), 50–59.

[ref86] Newton, R. U., Young, W. B., Kraemer, W. J., & Byrne, C. (2001). Effects of drop jump height and technique on ground reaction force with possible implication for injury. Research in Sports Medicine, 10(2), 83–93.

[ref87] Oliver, J. L., Barillas, S. R., Lloyd, R. S., Moore, I., & Pedley, J. (2021). External Cueing Influences Drop Jump Performance in Trained Young Soccer Players. Journal of Strength and Conditioning Research, 35(6), 1700–1706. 30676388 10.1519/JSC.0000000000002935

[ref88] Pedley, J. S., Lloyd, R. S., Read, P., Moore, I. S., & Oliver, J. L. (2017). Drop jump: A technical model for scientific application. Strength and Conditioning Journal, 39(5), 36–44.

[ref89] Peng, H. T. (2011). Changes in biomechanical properties during drop jumps of incremental height. Journal of Strength and Conditioning Research, 25(9), 2510–2518. 21869631 10.1519/JSC.0b013e318201bcb3

[ref90] Peng, H. T., Kernozek, T. W., & Song, C. Y. (2011). Quadricep and hamstring activation during drop jumps with changes in drop height. Physical Therapy in Sport, 12(3), 127–132. 21802039 10.1016/j.ptsp.2010.10.001

[ref91] Peng, H. T., Khuat, C. T., Kernozek, T. W., Wallace, B. J., Lo, S. L., & Song, C. Y. (2017). Optimum Drop Jump Height in Division III Athletes: Under 75% of Vertical Jump Height. International Journal of Sports Medicine, 38(11), 842–846. 28895621 10.1055/s-0043-114011

[ref92] Pereira, L., Winckler, C., Abad, C. C., Kobal, R., Kitamura, K., Veríssimo, A., Loturco, I. (2016). Power and Speed Differences Between Brazilian Paralympic Sprinters With Visual Impairment and Their Guides. Adapted Physical Activity Quarterly, 33(4), 311–323. 27874306 10.1123/APAQ.2015-0006

[ref93] Perttunen, J. O., Kyrolainen, H., Komi, P. V., & Heinonen, A. (2000). Biomechanical loading in the triple jump. Journal of Sports Science, 18(5), 363–370. 10.1080/026404100402421 10855682

[ref94] Prapavessis, H., McNair, P. J., Anderson, K., & Hohepa, M. (2003). Decreasing landing forces in children: the effect of instructions. Journal of Orthopaedic & Sports Physical Therapy, 33(4), 204–207. 12723677 10.2519/jospt.2003.33.4.204

[ref95] Pullen, B. J., Oliver, J. L., Lloyd, R. S., & Knight, C. J. (2022). Assessing athletic motor skill competencies in youths: a narrative review of movement competency screens. Strength and Conditioning Journal, 44(1), 95–110.

[ref96] Ramirez-Campillo, R., Alvarez, C., García-Pinillos, F., Sanchez-Sanchez, J., Yanci, J., Castillo, D., Izquierdo, M. (2018). Optimal Reactive Strength Index: Is It an Accurate Variable to Optimize Plyometric Training Effects on Measures of Physical Fitness in Young Soccer Players? Journal of Strength and Conditioning Research, 32(4), 885–893. 29389692 10.1519/JSC.0000000000002467

[ref97] Ramirez-Campillo, R., Castillo, D., Raya-Gonzalez, J., Moran, J., de Villarreal, E. S., & Lloyd, R. S. (2020). Effects of Plyometric Jump Training on Jump and Sprint Performance in Young Male Soccer Players: A Systematic Review and Meta-analysis. Sports Medicine, 50(12), 2125–2143. 32915430 10.1007/s40279-020-01337-1

[ref98] Rimmer, E., & Sleivert, G. (2000). Effects of a plyometrics intervention program on sprint performance. Journal of Strength and Conditioning Research, 14(3), 295–301.

[ref99] Saez de Villarreal, E., Requena, B., & Cronin, J. B. (2012). The effects of plyometric training on sprint performance: a meta-analysis. Journal of Strength and Conditioning Research, 26(2), 575–584. 22240550 10.1519/JSC.0b013e318220fd03

[ref100] Selmi, M. A., Sassi, R. H., Yahmed, M. H., Giannini, S., Perroni, F., & Elloumi, M. (2020). Normative Data and Physical Determinants of Multiple Sprint Sets in Young Soccer Players Aged 11-18 Years: Effect of Maturity Status. Journal of Strength and Conditioning Research, 34(2), 506–515. 30239457 10.1519/JSC.0000000000002810

[ref101] Sheppard, J. M., Dingley, A. A., Janssen, I., Spratford, W., Chapman, D. W., & Newton, R. U. (2011). The effect of assisted jumping on vertical jump height in high-performance volleyball players. Journal of Science and Medicine in Sport, 14(1), 85–89. 20829109 10.1016/j.jsams.2010.07.006

[ref102] Slimani, M., Chamari, K., Miarka, B., Del Vecchio, F. B., & Chéour, F. (2016). Effects of Plyometric Training on Physical Fitness in Team Sport Athletes: A Systematic Review. Journal of Human Kinetics, 53, 231–247. 28149427 10.1515/hukin-2016-0026 PMC5260592

[ref103] Sole, C. J. (2018). Plyometric training. In A. Turner & P. Comfort (Eds.), Advanced strength and conditioning: An evidence-based approach (pp. 274–290). Abingdon, NY: Routledge.

[ref104] Sotiropoulos, K., Smilios, I., Barzouka, K., Christou, M., Bogdanis, G., Douda, H., & Tokmakidis, S. P. (2023). Effects of Drop Jump Training from Different Heights and Weight Training on Vertical Jump and Maximum Strength Performance in Female Volleyball Players. Journal of Strength and Conditioning Research, 37(2), 423–431. 35836281 10.1519/JSC.0000000000004272

[ref105] Struzik, A., Juras, G., Pietraszewski, B., & Rokita, A. (2016). Effect of drop jump technique on the reactive strength index. Journal of Human Kinetics, 52, 157–164. 28149403 10.1515/hukin-2016-0003 PMC5260527

[ref106] Turner, A. N., & Jeffreys, I. (2010). The stretch-shortening cycle: Proposed mechanisms and methods for enhancement. Strength and Conditioning Journal, 32(4), 87–99.

[ref107] Turner, A. P., Bellhouse, S., Kilduff, L. P., & Russell, M. (2015). Postactivation potentiation of sprint acceleration performance using plyometric exercise. Journal of Strength and Conditioning Research, 29(2), 343–350. 25187244 10.1519/JSC.0000000000000647

[ref108] Vanrenterghem, J., Lees, A., & Clercq, D. D. (2008). Effect of forward trunk inclination on joint power output in vertical jumping. Journal of Strength and Conditioning Research, 22(3), 708–714. 18438250 10.1519/JSC.0b013e3181636c6c

[ref109] Vescovi, J. D., Canavan, P. K., & Hasson, S. (2008). Effects of a plyometric program on vertical landing force and jumping performance in college women. Physical Therapy in Sport, 9(4), 185–192. 19083719 10.1016/j.ptsp.2008.08.001

[ref110] Waller, M., Shim, A., & Piper, T. (2019). Strength and conditioning off-season programming for high school swimmers. Strength and Conditioning Journal, 41(5), 79–85.

[ref111] Waller, M. A., Gersick, M. J., Townsend, R. J., & Ford, C. N. (2014). Strength and conditioning preparation for the transitional track and field thrower. Strength and Conditioning Journal, 36(6), 71–78.

[ref112] Weldon, A., Duncan, M. J., Turner, A., Christie, C. J., & Pang, C. M. C. (2021). Contemporary practices of strength and conditioning coaches in professional cricket. International Journal of Sports Science and Coaching, 16(3), 585–600.

[ref113] Weldon, A., Duncan, M. J., Turner, A., LaPlaca, D., Sampaio, J., & Christie, C. J. (2022a). Practices of Strength and Conditioning Coaches: A Snapshot From Different Sports, Countries, and Expertise Levels. Journal of Strength and Conditioning I, 36(5), 1335–1344. 10.1519/JSC.0000000000003773 33298715

[ref114] Weldon, A., Duncan, M. J., Turner, A., Lockie, R. G., & Loturco, I. (2022b). Practices of strength and conditioning coaches in professional sports: a systematic review. Biology of Sport, 39(3), 715–726. 35959337 10.5114/biolsport.2022.107480 PMC9331342

[ref115] Whelan, N., Kenny, I. C., & Harrison, A. J. (2016). An insight into track and field coaches’ knowledge and use of sprinting drills to improve performance. International Journal of Sports Science and Coaching, 11(2), 182–190.

[ref116] Wing, C. (2018). In-season strength and power training considerations for professional soccer teams competing within national level competitions. Strength and Conditioning Journal, 40(3), 12–22.

[ref117] Young, W. B. (1992). Plyometrics: Sprint bounding and the sprint bound index. Strength and Conditioning Journal, 14(4), 18–22.

[ref118] Young, W. B. (2006). Transfer of strength and power training to sports performance. International Journal of Sports Physiology and Performance, 1(2), 74–83. 19114741 10.1123/ijspp.1.2.74

[ref119] Young, W. B., Benton, D., & Pryor, J. (2001). Resistance training for short sprints and maximum-speed sprints. Strength and Conditioning Journal, 23(2), 7.

[ref120] Young, W. B., Wilson, G. J., & Byrne, C. (1999). A comparison of drop jump training methods: effects on leg extensor strength qualities and jumping performance. International Journal of Sports Medicine, 20(5), 295–303. 10452226 10.1055/s-2007-971134

[ref121] Zabaloy, S., Tondelli, E., Pereira, L. A., Freitas, T. T., & Loturco, I. (2022). Training and testing practices of strength and conditioning coaches in Argentinian Rugby Union. International Journal of Sports Science and Coaching, 17(6), 1331–1344.

